# Negative cancer beliefs, recognition of cancer symptoms and anticipated time to help-seeking: an international cancer benchmarking partnership (ICBP) study

**DOI:** 10.1186/s12885-018-4287-8

**Published:** 2018-04-02

**Authors:** Anette Fischer Pedersen, Lindsay Forbes, Kate Brain, Line Hvidberg, Christian Nielsen Wulff, Magdalena Lagerlund, Senada Hajdarevic, Samantha L. Quaife, Peter Vedsted

**Affiliations:** 1 Research Unit for General Practice and Research Centre for Cancer Diagnosis in Primary Care (CaP), Aarhus University, Bartholins Allé 2, 8000 Aarhus C, Denmark; 2 King’s College London Promoting Early Cancer Presentation Group, Capital House, 42 Weston Street, London, SE1 3QD UK; 3 Cochrane Institute of Primary Care and Public Health, Neuadd Meirionydd, School of Medicine, Cardiff University, Heath Park, Cardiff, CF14 4YS UK; 4 Department of Oncology, Aarhus University Hospital, Norrebrogade 44, 8000 Aarhus C, Denmark; 5 Department of Learning, Informatics, Management and Ethics (LIME), Karolinska Institutet, Tomtebodavägen 18A, Stockholm, Sweden; 6 Department of Nursing, Umeå University, SE-901 87 Umeå, Sweden; 7 Health Behaviour Research Centre, Department of Epidemiology and Public Health, University College London, Gower Street, WC1E 6BT, London, UK

**Keywords:** Behavioural medicine, Primary health care, Surveys and questionnaires, Telephone

## Abstract

**Background:**

Understanding what influences people to seek help can inform interventions to promote earlier diagnosis of cancer, and ultimately better cancer survival. We aimed to examine relationships between negative cancer beliefs, recognition of cancer symptoms and how long people think they would take to go to the doctor with possible cancer symptoms (anticipated patient intervals).

**Methods:**

Telephone interviews of 20,814 individuals (50+) in the United Kingdom, Australia, Canada, Denmark, Norway and Sweden were carried out using the Awareness and Beliefs about Cancer Measure (ABC). ABC included items on cancer beliefs, recognition of cancer symptoms and anticipated time to help-seeking for cough and rectal bleeding. The anticipated time to help-seeking was dichotomised as over one month for persistent cough and over one week for rectal bleeding.

**Results:**

Not recognising persistent cough/hoarseness and unexplained bleeding as cancer symptoms increased the likelihood of a longer anticipated patient interval for persistent cough (OR = 1.66; 95%CI = 1.47–1.87) and rectal bleeding (OR = 1.90; 95%CI = 1.58–2.30), respectively. Endorsing four or more out of six negative beliefs about cancer increased the likelihood of longer anticipated patient intervals for persistent cough and rectal bleeding (OR = 2.18; 95%CI = 1.71–2.78 and OR = 1.97; 95%CI = 1.51–2.57). Many negative beliefs about cancer moderated the relationship between not recognising unexplained bleeding as a cancer symptom and longer anticipated patient interval for rectal bleeding (*p* = 0.005).

**Conclusions:**

Intervention studies should address both negative beliefs about cancer and knowledge of symptoms to optimise the effect.

**Electronic supplementary material:**

The online version of this article (10.1186/s12885-018-4287-8) contains supplementary material, which is available to authorized users.

## Background

Diagnosis of cancer at an early stage is important to optimise the outcomes of cancer [1]. A study of more than 2000 patients with 15 different cancers showed that 21% had delayed symptomatic presentation for more than three months [2]. Therefore, improvement of our understanding of individuals’ decision to seek medical help for symptoms that could be a sign of cancer is very important.

The patient interval is defined as the time period between an individual’s first discovery of a change in the body and the first consultation with a healthcare professional, often the general practitioner [1]. Cognitive factors such as knowledge about disease and symptoms seem to play a role in decision-making about healthcare seeking [3–5]. For instance, cancer patients who did not perceive their initial symptoms as serious were twice as likely to postpone help-seeking for at least three months [2]. Nevertheless, there is often a gap between knowledge and behaviour, with knowledge about cancer symptoms not entirely predictive of help-seeking behaviour [6, 7]. The relationship between negative cancer beliefs (NCBs) and help-seeking for a symptom which may be a sign of cancer has been under-researched.

**Fig. 1:**
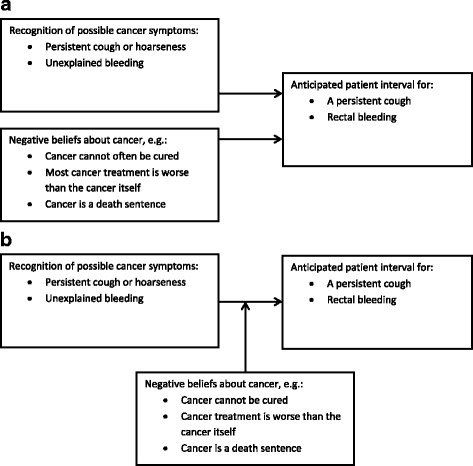
Models of negative cancer beliefs as either independent variable or effect moderator. **a** Independent effects of negative beliefs about cancer and recognition of cancer symptoms on anticipated patient intervals. **b** Negative beliefs about cancer as an effect moderator of the association between recognition of cancer symptoms and length of the anticipated patient interval

The aim of this paper was to explore the influence of NCBs on the likelihood of long anticipated patient intervals for persistent cough and rectal bleeding. Persistent cough and rectal bleeding may be signs of lung cancer and colorectal cancer, respectively, and these two cancers strike both men and women. They are two of the most common cancers, yet their symptoms may be perceived as less alarming or less specific to cancer than “classic” symptoms such as a lump. First, we examined the association between NCBs about cancer and longer anticipated patient intervals for persistent cough and rectal bleeding while adjusting for symptom recognition (see Fig. 1a). Second, we examined whether NCBs about cancer have a moderating influence on the association between not recognising persistent cough/hoarseness or unexplained bleeding as possible signs of cancer and a long anticipated patient interval for persistent cough and rectal bleeding, respectively (see Fig. 1b).

## Methods

Data were used from a survey conducted as part of the International Cancer Benchmarking Partnership (ICBP) which was initiated to study international variation in cancer survival. Module 2 of the ICBP measured population awareness and beliefs about cancer [8]. Data were collected from May to September 2011 [9] in six countries: the United Kingdom (England, Wales and Northern Ireland), Australia (New South Wales and Victoria), Canada, Denmark, Norway and Sweden.

Computer-assisted telephone interviews were carried out by the research market company Ipsos MORI. All men and women aged 50 years or more living in private households and able to understand the official language of the country were eligible. The researchers aimed to achieve a sample size of 2000 respondents in each country or jurisdiction, and a total of 20,814 respondents participated. Data collection and response rates have been described in detail elsewhere [9–11].

NCBs, recognition of cancer symptoms and anticipated help-seeking were assessed by the internationally developed and validated Awareness and Beliefs about Cancer Measure (ABC) [12].

### Dependent variable: anticipated time to help-seeking for persistent cough and rectal bleeding

Four items from the ABC assessed the anticipated patient interval for symptoms that could be signs of cancer. In the present study, lung cancer and colorectal cancer were the two target cancers and the anticipated patient intervals for these two cancers were assessed by asking the respondents how long they would wait before seeking help after experiencing a persistent cough and rectal bleeding, respectively. No standardised and internationally accepted guidelines are available about when to seek help for these symptoms. As presented in Table 1, anticipated patient intervals of more than one month for persistent cough and of more than one week for rectal bleeding were categorised as “long”. These cut-offs were chosen for two reasons. First they ensured that we had enough subjects in our “long” and “short” patient interval categories. Second, cut-offs appeared reasonable as everyone experiences a cough now and then and the duration of the symptom plays an important role when determining whether the cough needs medical attention. On the contrary, rectal bleeding happening more than once should always cause concern.

**Table 1:** Responses to questions about anticipated patient intervals and symptom awareness and how they were classified

Dependent variables: Anticipated patient intervals				
	How long would it take you to go to the doctor from the first time you noticed a persistent cough?		How long would it take you to go to the doctor from the first time you noticed rectal bleeding?	
Response categories:	N (%)	Classification	N (%)	Classification
I would go as soon as I noticed	2669 (12.8)	Short pt. interval	12,617 (60.6)	Short pt. interval
Up to 1 week	3750 (18.0)	Short pt. interval	5017 (24.1)	Short pt. interval
Over 1 up to 2 weeks	4029 (19.4)	Short pt. interval	1323 (6.4)	Long pt. interval
Over 2 up to 3 weeks	3001 (14.4)	Short pt. interval	518 (2.5)	Long pt. interval
Over 3 up to 4 weeks	2557 (12.3)	Short pt. interval	357 (1.7)	Long pt. interval
More than a month	3267 (15.7)	Long pt. interval	488 (2.3)	Long pt. interval
I would not contact a/my doctor	844 (4.1)	Long pt. interval	171 (0.8)	Long pt. interval
I would go to another HCP	320 (1.5)	Missing	141 (0.7)	Missing
Don’t know	355 (1.7)	Missing	164 (0.8)	Missing
Don’t want to answer	22 (0.1)	Missing	18 (0.1)	Missing
*Independent variables: Recognition of symptoms as cancer symptoms*				
	Do you think persistent cough or hoarseness could be a warning sign for cancer?		Do you think unexplained bleeding could be a warning sign for cancer?	
Response categories:	N (%)	Classification	N (%)	Classification
Yes	15,155 (72.8)	Recognition	18,187 (87.4)	Recognition
No	4951 (23.8)	Non-recognition	2018 (9.7)	Non-recognition
Don’t know	693 (3.3)	Missing	598 (2.9)	Missing
Don’t want to answer	15 (7.2)	Missing	11 (0.0)	Missing
*Independent variable/moderator: Negative cancer beliefs (NCBs)*				
*Answers classified as NCBs are highlighted in bold.*				
*The answers “don’t know” and “don’t want to answer” were classified as missing.*				
	Strongly disagree or tend to disagree		Strongly agree or tend to agree	Don’t know or refused
	N (%)		N (%)	N (%)
People with cancer can expect to continue with normal activities	**1936 (9.3)**		18,187 (87.4)	691 (3.3)
Cancer can often be cured	**1853 (8.9)**		18,502 (88.9)	459 (2.2)
Going to the doctor as quickly as possible after noticing a symptom of cancer could increase the chances of surviving	**394 (1.9)**		20,310 (97.6)	110 (0.5)
Cancer treatment is worse than the cancer itself	7196 (34.6)		**11,082 (53.2)**	2536 (12.2)
I would not want to know if I have cancer	18,004 (86.5)		**2337 (11.2)**	473 (2.3)
A diagnosis of cancer is a death sentence	14,995 (72.0)		**5110 (24.6)**	709 (3.4)

HCP = Healthcare professional; pt. = patient

### Independent variable: recognition of persistent cough or hoarseness and unexplained bleeding as cancer symptoms

The ABC included 11 possible cancer symptoms and respondents were asked to determine whether they thought that each symptom could be a warning sign of cancer. The warning signs most closely related to the two target cancers were persistent cough or hoarseness and unexplained bleeding. Response options and coding are shown in Table 1.

### Independent variable and hypothesised moderator: negative cancer beliefs (NCBs)

Six statements examined respondents’ beliefs about cancer. The wording, response options and coding are shown in Table 1. The number of NCBs endorsed was summed for each respondent. As the proportion of respondents declaring five or six NCBs was limited (0.5%), the survey data from these respondents were grouped with the survey data from respondents declaring four NCBs to ensure sufficient statistical power.

### Demographic characteristics

Data were collected on sex, age (categorised as 50 to 59 years, 60 to 69 years, 70 to 79 years and 80 years or older), marital status (categorised as married/cohabiting vs. single/divorced/separated/widowed), highest level of education (categorised as no university degree vs. university degree), experience of cancer themselves and/or in family/friend (categorised as yes/no), country and smoking status (categorised as never/former vs. current). Smokers have been shown to declare more NCBs than non-smokers [13].

### Statistics

The associations between non-recognition of persistent cough or hoarseness and unexplained bleeding as cancer symptoms, number of NCBs about cancer and long anticipated patient intervals for persistent cough and rectal bleeding, respectively, were examined in two separate logistic regression analyses. Odds ratios (ORs) were calculated as measures of association. In the adjusted analyses, both non-recognition of symptom and number of NCBs were included as independent variables and sex, age, marital status, highest level of education, smoking status, experience of cancer and country were included as co-variables. As a sensitivity analysis, the logistic regression analyses were repeated with the answer “don’t know” to the questions about whether persistent cough or hoarseness and unexplained bleeding could be signs of cancer coded as “non-recognition” instead of missing. To test for a moderating effect of NCBs on the association between non-recognition of the symptoms and length of anticipated patient intervals, two interaction terms were computed: non-recognition of persistent cough or hoarseness as a cancer symptom multiplied with the number of NCBs and non-recognition of unexplained bleeding as a cancer symptom multiplied with the number of NCBs. These two interaction terms were included in two separate logistic regression analyses which also included independent and demographic variables. The likelihood-ratio (LR) test was used to determine whether the models with and without the interaction terms were statistically significantly different. In case of a statistically significant interaction, predictive margins would be used to visualise the effect and to assist interpretation (https://www.cscu.cornell.edu/news/statnews/stnews84.pdf). *P*-values of 5% or less were considered statistically significant. Data were analysed using STATA 13.1.

## Results

Demographic characteristics and proportion of respondents with none to four or more NCBs are shown in Table 2 (see additional file 1 for these results reported for each country).

**Table 2:** Description of the sample

	All	Females	Males
	*N* = 20,814 (100%)	*N* = 12,456 (59.8%)	*N* = 8358 (40.2%)
Age group, n (%)			
50–59 years	7375 (35.4)	4414 (35.4)	2961 (35.4)
60–69 years	7510 (36.1)	4416 (35.5)	3094 (37.0)
70–79 years	4234 (20.3)	2564 (20.6)	1670 (20.0)
≥ 80 years	1695 (8.1)	1062 (8.5)	633 (7.6)
Marital status			
Cohabiting	12,665 (60.9)	6747 (54.2)	5918 (70.8)
Single	8082 (38.8)	5665 (45.5)	2417 (28.9)
Education			
No university degree	14,334 (68.9)	8911 (71.5)	5423 (64.9)
University degree	6331 (30.4)	3448 (27.7)	2883 (34.5)
Smoking status			
Not current smoker	17,600 (84.6)	10,605 (85.1)	6995 (83.7)
Current smoker	3207 (15.4)	1847 (14.8)	1360 (16.3)
Experience of cancer (self and/or family/friend)			
Yes	17,157 (82.4)	10,698 (85.9)	6459 (77.3)
No	3629 (17.4)	1745 (14.0)	1884 (22.5)
Anticipated interval for persistent cough			
Short	16,006 (76.9)	9578 (76.9)	6428 (76.9)
Long (> 1 month)	4111 (19.8)	2466 (19.8)	1645 (19.7)
Anticipated interval for rectal bleeding			
Short	17,634 (84.7)	10,588 (85.0)	7046 (84.3)
Long (> 1 week)	2857 (13.7)	1675 (13.5)	1182 (14.1)
Recognition of persistent cough or hoarseness as cancer symptom			
Yes	15,155 (72.8)	9501 (76.3)	5654 (67.7)
No	4951 (23.8)	2558 (20.5)	2393 (28.6)
Recognition of unexplained bleeding as cancer symptom			
Yes	18,187 (87.4)	11,157 (89.6)	7030 (84.1)
No	2018 (9.7)	1005 (8.1)	1013 (12.1)
Number of negative cancer beliefs (coded as)			
0 (0)	6427 (30.9)	3420 (27.5)	3007 (36.0)
1 (1)	8412 (40.4)	5225 (42.0)	3187 (38.1)
2 (2)	4180 (20.1)	2682 (21.5)	1498 (17.9)
3 (3)	1347 (6.5)	849 (6.8)	498 (6.0)
4–6 (> 4)	448 (2.2)	280 (2.3)	168 (2.0)

Sums vary because of missing data

Not recognising persistent cough or hoarseness as a possible sign of cancer (OR = 1.66, 95% CI = 1.47–1.87) and a high number of NCBs (OR_≥ 4 NCBs_ = 2.18, 95% CI = 1.71–2.78) were independently associated with an increased likelihood of a longer anticipated patient interval for persistent cough (Table 3). Not recognising unexplained bleeding as a possible sign of cancer (OR = 1.90, 95% CI = 1.58–2.30) and a high number of NCBs (OR_≥ 4 NCBs_ = 1.97, 95% CI = 1.51–2.57) were independently associated with an increased likelihood of a longer anticipated patient interval for rectal bleeding (Table 4). In both models, there was a dose-response relationship between the number of NCBs and the likelihood of a longer anticipated patient interval.

**Table 3:** Associations between recognition of persistent cough or hoarseness as cancer symptom, negative beliefs about cancer and long anticipated patient interval for persistent cough (n_adjusted_ = 19,277)

	Unadjusted				Adjusted*		
	Outcome: long anticipated patient interval for cough (> 1 month)				Outcome: long anticipated patient interval for cough (> 1 month)		
	Proportion with long interval (%)	OR	95% CI	*P*-value	OR	95% CI	*P*-value
Recognition of persistent cough or hoarseness as cancer symptom							
Yes	18.5	Ref.			Ref.		
No	26.7	1.60	1.49–1.73	< 0.001	1.66	1.47–1.87	< 0.001
Number of negative beliefs about cancer							
0	19.8	Ref.			Ref.		
1	19.3	0.97	0.89–1.05	0.453	0.97	0.89–1.06	0.522
2	20.7	1.05	0.95–1.16	0.312	1.06	0.95–1.19	0.305
3	24.5	1.31	1.14–1.51	< 0.001	1.33	1.13–1.57	0.001
≥ 4	36.3	2.31	1.87–2.84	< 0.001	2.18	1.71–2.78	< 0.001
Age group							
50–59 years	23.1	Ref.			Ref.		
60–69 years	21.3	0.90	0.84–0.98	0.012	0.93	0.86–1.01	0.087
70–79 years	17.0	0.68	0.62–0.76	< 0.001	0.75	0.67–0.83	< 0.001
≥ 80 years	13.1	0.50	0.43–0.59	< 0.001	0.57	0.48–0.67	< 0.001
Sex							
Female	20.5	Ref.			Ref.		
Male	20.4	0.99	0.93–1.07	0.865	0.93	0.86–1.00	0.042
Marital status							
Cohabiting	21.1	Ref.			Ref.		
Single	19.3	0.89	0.83–0.96	0.002	0.93	0.86–1.00	0.057
Education							
No university degree	19.6	Ref.			Ref.		
University degree	22.3	1.18	1.09–1.27	< 0.001	1.24	1.15–1.34	< 0.001
Smoking status							
Not current smoker	19.4	Ref.			Ref.		
Current smoker	26.1	1.47	1.34–1.60	< 0.001	1.38	1.26–1.52	< 0.001
Experience of cancer (self and/or family/friend)							
Yes	20.7	Ref.			Ref.		
No	19.0	0.90	0.82–0.98	0.020	0.85	0.77–0.93	0.001
Country							
UK	22.5	Ref.			Ref.		
Australia	16.9	0.70	0.63–0.77	< 0.001	0.70	0.63–0.78	< 0.001
Canada	18.8	0.79	0.72–0.88	< 0.001	0.74	0.67–0.83	< 0.001
Denmark	21.3	0.93	0.82–1.05	0.229	0.88	0.78–1.00	0.053
Norway	24.9	1.14	1.01–1.28	0.031	1.10	0.97–1.24	0.137
Sweden	18.2	0.77	0.67–0.87	< 0.001	0.72	0.64–0.83	< 0.001
*Test of moderation*							
Negative beliefs x Non-recognition of persistent cough or hoarseness					0.99	0.92–1.07	0.831
*Likelihood-ratio test*							
LR chi^2^							0.05
P-value							0.831

*All independent variables were included in the same model

**Table 4:** Associations between recognition of unexplained bleeding as cancer symptom, negative beliefs about cancer and long anticipated patient interval for rectal bleeding (n_adjusted_ = 19,695)

	Unadjusted				Adjusted*		
	Outcome: long anticipated patient interval for rectal bleeding (> 1 week)				Outcome: long anticipated patient interval for rectal bleeding (> 1 week)		
	Proportion with long interval (%)	OR	95% CI	*P*-value	OR	95% CI	*P*-value
Recognition of unexplained bleeding as cancer symptom							
Yes	13.3	Ref.			Ref.		
No	19.7	1.59	1.41–1.79	< 0.001	1.90	1.58–2.30	< 0.001
Number of negative beliefs about cancer							
0	13.0	Ref.			Ref.		
1	13.8	1.07	0.98–1.18	0.147	1.11	1.00–1.22	0.051
2	14.4	1.13	1.01–1.27	0.034	1.21	1.07–1.37	0.002
3	15.8	1.26	1.06–1.48	0.007	1.41	1.18–1.69	< 0.001
≥ 4	20.3	1.71	1.34–2.18	< 0.001	1.97	1.51–2.57	< 0.001
Age group							
50–59 years	16.6	Ref.			Ref.		
60–69 years	14.0	0.82	0.75–0.90	< 0.001	0.82	0.75–0.90	< 0.001
70–79 years	11.4	0.65	0.58–0.73	< 0.001	0.67	0.59–0.76	< 0.001
≥ 80 years	8.5	0.46	0.39–0.56	< 0.001	0.51	0.41–0.62	< 0.001
Sex							
Female	13.7	Ref.			Ref.		
Male	14.4	1.06	0.98–1.15	0.152	1.03	0.95–1.12	0.495
Marital status							
Cohabiting	14.3	Ref.			Ref.		
Single	13.5	0.93	0.86–1.01	0.103	1.08	0.99–1.18	0.086
Education							
No university degree	12.9	Ref.			Ref.		
University degree	16.4	1.33	1.22–1.44	< 0.001	1.29	1.18–1.41	< 0.001
Smoking status							
Not current smoker	14.0	Ref.			Ref.		
Current smoker	13.9	1.00	0.90–1.12	0.990	0.91	0.81–1.02	0.111
Experience of cancer (self and/or family/friend)							
Yes	14.2	Ref.			Ref.		
No	12.6	0.87	0.78–0.97	0.010	0.85	0.76–0.95	0.006
Country							
UK	13.6	Ref.			Ref.		
Australia	8.6	0.59	0.52–0.68	< 0.001	0.60	0.52–0.68	< 0.001
Canada	15.1	1.13	1.01–1.27	0.033	1.04	0.93–1.17	0.480
Denmark	14.6	1.08	0.94–1.25	0.270	1.00	0.86–1.16	0.990
Norway	19.4	1.52	1.34–1.74	< 0.001	1.46	1.27–1.68	< 0.001
Sweden	17.1	1.31	1.14–1.50	< 0.001	1.22	1.06–1.41	0.005
*Test of moderation*							
Negative beliefs x Non-recognition of unexplained bleeding					0.84	0.75–0.95	0.005
*Likelihood-ratio test*							
LR chi^2^							7.97
P-value							0.005

*All independent variables were included in the same model

The results of the sensitivity analysis (the answer “don't know” coded as “non-recognition” of symptom instead of missing) confirmed a statistically independent influence of non-recognition of symptoms (OR_persistent cough_ = 1.57, 95% CI = 1.40–1.77; OR_rectal bleeding_ = 1.70, 95% CI = 1.43–2.01) and number of NCBs (OR_persistent cough_ = 2.27, 95% CI = 1.79–2.89; OR_rectal bleeding_ = 1.98, 95% CI = 1.53–2.58) on the likelihood of a long anticipated patient interval for persistent cough and rectal bleeding (adjusted analyses, data not shown).

**Fig. 2:**
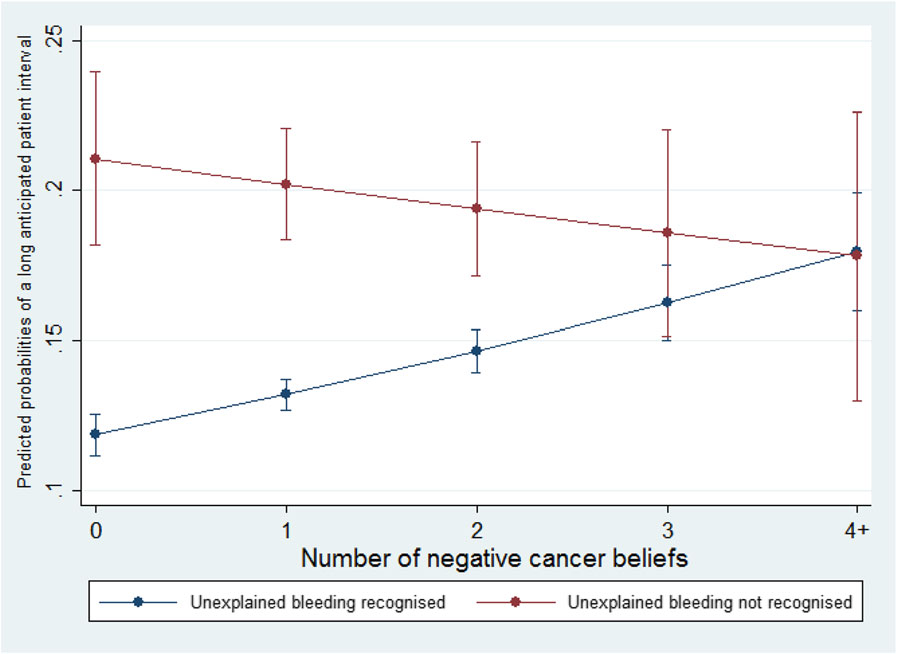
Predicted probabilities of a long anticipated patient interval with 95% CIs for respondents who recognised or did not recognise unexplained bleeding as cancer symptom and with various levels of negative cancer beliefs

No statistically significant interaction effect of NCBs and not recognising cough or hoarseness on the likelihood of a long anticipated patient interval for persistent cough was revealed in the moderation analysis (Table 3). However, the moderation analysis revealed a significant interaction of NCBs with non-recognition of unexplained bleeding on the likelihood of a long anticipated patient interval for rectal bleeding, and the likelihood-ratio test confirmed that the statistical model with the interaction term was significantly different from the model without the interaction term (LR chi^2^ = 7.97, *p* = 0.005; Table 4). In Fig. 2, the predicted probabilities of reporting a long anticipated patient interval for rectal bleeding across different levels of NCBs are depicted. The predicted probability of a long anticipated patient interval for those who recognised unexplained bleeding as a possible cancer symptom increased with number of NCBs. For those reporting four or more NCBs, the predicted probability of a long anticipated patient interval for rectal bleeding was the same regardless of whether they recognised unexplained bleeding as a cancer symptom or not. For those who did not recognise unexplained bleeding as a cancer symptom, the predicted probabilities for a long anticipated patient interval for rectal bleeding decreased with increasing number of NCBs.

## Discussion

### Main findings

Not recognising persistent cough/hoarseness and unexplained bleeding as possible signs of cancer and number of NCBs were independently associated with an increased likelihood of longer anticipated patient intervals for persistent cough and rectal bleeding, respectively. There was a dose-response relationship between number of NCBs and longer anticipated patient intervals for persistent cough and rectal bleeding. Hence, a high level of NCBs may act as a barrier to help-seeking when experiencing a symptom that could be a sign of cancer. Furthermore, we found that number of NCBs was an effect moderator as when respondents reported four or more NCBs, the risk of a long anticipated patient interval for rectal bleeding would be the same whether the respondent had recognised unexplained bleeding as a cancer symptom or not. Number of NCBs could only be documented as an effect moderator in the model of bleeding and not in the model of cough. Respondents estimated that they would wait longer before seeking help for a persisting cough than for rectal bleeding and in line with this finding, respondents in another survey seldom recognised cough or hoarseness as a cancer symptom [14]. If respondents do not see persistent cough as a “red flag” symptom in the same way they see rectal bleeding, it seems plausible that the role of NCBs may be different in the two models. The moderating potential of NCBs may only come into play when the symptom is experienced as serious and the symptom recognition thereby causes anxiety [15].

We find it difficult to explain why the probability of a long anticipated patient interval for rectal bleeding decreased with increasing number of NCBs among those who did not recognise unexplained bleeding as a cancer symptom. The number of respondents who did not recognise unexplained bleeding as a cancer symptom was relatively small compared to those who did and therefore, when subdividing the group based on number of NCBs, the confidence intervals became rather wide as was evident in Fig. 2 and results from this analysis should be interpreted with caution.

### Strengths and limitations

Among the strengths of this study was the use of a population-representative sample and that we used an internationally validated questionnaire for assessing recognition of cancer symptoms and NCBs outcomes [12]. A potential weakness is that the anticipated help-seeking interval was hypothetical and might not mirror the response if a person experienced the symptom in reality. The majority of respondents (61%) reported that they would seek help immediately if they experienced rectal bleeding, but a review of 38 studies examining patient intervals in colorectal cancer showed that the actual median patient interval ranged from 7 days to 5 months [16]. This suggests that at least some of the participants in our study have underestimated how long they would actually wait before seeking help. Further, since the present data were cross-sectional, the causality of associations cannot be determined.

### Comparisons with existing literature

Several other studies have found that lack of knowledge of cancer symptoms is associated with longer anticipated patient intervals [5] and actual patient intervals [2–4]. The influence of NCBs on length of the patient interval has been less frequently studied. Qualitative studies have found that negative expectations of the health care professional make patients with potentially malignant oral symptoms more likely to postpone help-seeking [17], that older women (≥ 65 years) were deterred from seeking help because of negative expectations to surgical and medical treatments [18] and that poor confidence in the healthcare system was a reason for not seeking help when experiencing cancer alarm symptoms [19]. Further, a review of 32 qualitative studies revealed that a fatalistic attitude to cancer was one of the main reasons for delaying help-seeking as well as interpreting symptoms as benign and self-limiting [20].

The Extended Parallel Process Model (EPPM) [21, 22] may be useful for explaining the moderating effect of NCBs on the increased likelihood of longer anticipated patient intervals for potential colorectal symptoms. Thus, recognition of a symptom as a possible sign of cancer may give rise to fear, which has been identified as a barrier to help-seeking as well as a factor which could promote prompt help-seeking for cancer symptoms [19, 23]. According to the EPPM, fear will induce protective danger control processes (e.g. fast help-seeking) only if the individual believes that s/he is able to deal with the threat [21, 24]. If NCBs about the disease and its treatment are salient and the individual believes that no action will be effective dealing with the disease, the fear will induce fear control processes such as denial and downplay of worrisome symptoms, meaning that help-seeking may be postponed.

## Conclusion

It is often assumed that patients with sufficient knowledge about possible cancer symptoms would engage in appropriate help-seeking [6]. The results of the present study support this notion, as people who recognised cancer symptoms reported shorter anticipated patient intervals compared to people who did not recognise these symptoms as possible signs of cancer. Meanwhile, a high number of NCBs also increased the likelihood of long anticipated patient intervals, and the association between recognition of unexplained bleeding as a cancer symptom and the intention to seek help quickly for rectal bleeding was only seen in respondents who reported few NCBs. Therefore, it is equally important that NCBs are addressed in interventions designed to shorten patient intervals.

## Additional file

Additional file 1: Description of sample countrywise. (DOC 81 kb)

## Abbreviations

ABC: Awareness and beliefs about cancer Measure
CI: Confidence interval
EPPM: Extended Parallel Process Model
ICBP: International cancer benchmarking partnership
NCB: Negative cancer belief
