# Supplementary material for: Negative cancer beliefs, recognition of cancer symptoms and anticipated time to help-seeking: an international cancer benchmarking partnership (ICBP) study
Source: BMC Cancer. 2018 Apr 2;18:363. doi: 10.1186/s12885-018-4287-8 (PMC5879768; doi:10.1186/s12885-018-4287-8)
Supplement: Supplementary file 1 — Description of sample countrywise. (DOC 81 kb) [file 12885_2018_4287_MOESM1_ESM.doc]

***Additional file:*** *Description of sample populations. Sums vary because of missing data.*

|  | **All**  **N = 20814** | **UK**  **N = 6900** | **Australia**  **N = 3959** | **Canada**  **N = 3907** | **Denmark**  **N = 2000** | **Norway**  **N = 2009** | **Sweden**  **N = 2039** |
| --- | --- | --- | --- | --- | --- | --- | --- |
| Age group, n (%) |  |  |  |  |  |  |  |
| 50-59 years | 7375 (35.4) | 2333 (33.8) | 1244 (31.4) | 1708 (43.7) | 746 (37.3) | 711 (35.4) | 633 (31.0) |
| 60-69 years | 7510 (36.1) | 2519 (36.5) | 1297 (32.8) | 1314 (33.6) | 764 (38.2) | 780 (38.8) | 836 (41.0) |
| 70-79 years | 4234 (20.3) | 1464 (21.2) | 892 (22.5) | 682 (17.5) | 392 (19.6) | 379 (18.9) | 425 (20.8) |
| > 80 years | 1695 (8.1) | 584 (8.5) | 526 (13.3) | 203 (5.2) | 98 (4.9) | 139 (6.9) | 145 (7.1) |
| Sex, n (%)… |  |  |  |  |  |  |  |
| Female | 12456 (59.8) | 4278 (62.0) | 2497 (63.1) | 2573 (65.9) | 1065 (53.3) | 932 (46.4) | 1111 (54.5) |
| Male | 8358 (40.2) | 2622 (38.0) | 1462 (36.9) | 1334 (34.1) | 935 (46.8) | 1077 (53.6) | 928 (45.5) |
| Marital status |  |  |  |  |  |  |  |
| Cohabiting | 12665 (60.9) | 3762 (54.5) | 2182 (55.1) | 2322 (59.4) | 1514 (75.7) | 1453 (72.3) | 1432 (70.2) |
| Single | 8082 (38.8) | 3109 (45.1) | 1760 (44.5) | 1571 (40.2) | 484 (24.2) | 553 (27.5) | 605 (29.7) |
| Education |  |  |  |  |  |  |  |
| No university degree | 14334 (68.9) | 5264 (76.3) | 2645 (66.8) | 2639 (67.6) | 1412 (70.6) | 1072 (53.4) | 1302 (63.9) |
| University degree | 6331 (30.4) | 1552 (22.5) | 1286 (32.5) | 1255 (32.1) | 582 (29.1) | 927 (46.1) | 729 (35.8) |
| Smoking status |  |  |  |  |  |  |  |
| Never-smokers | 8820 (42.4) | 3141 (45.5) | 1918 (48.5) | 1500 (38.4) | 673 (33.7) | 723 (36.0) | 865 (42.4) |
| Current smokers | 3207 (15.4) | 1043 (15.1) | 413 (10.4) | 648 (16.6) | 452 (22.6) | 372 (18.5) | 279 (13.7) |
| Former-smokers | 8771 (42.1) | 2713 (39.3) | 1624 (41.0) | 1754 (44.9) | 874 (43.7) | 912 (45.4) | 894 (43.9) |
| Experience of cancer (self and/or family/friend) |  |  |  |  |  |  |  |
| Yes | 17157 (82.4) | 5533 (80.2) | 3269 (82.6) | 3290 (84.2) | 1705 (85.3) | 1696 (84.4) | 1664 (81.6) |
| No | 3629 (17.4) | 1352 (19.6) | 684 (17.3) | 612 (15.7) | 295 (14.8) | 312 (15.5) | 373 (18.3) |
| Anticipated interval for persistent cough |  |  |  |  |  |  |  |
| Short | 16006 (76.9) | 5150 (74.6) | 3179 (80.3) | 3044 (77.9) | 1541 (77.1) | 1458 (72.6) | 1634 (80.1) |
| Long (> 1 month) | 4111 (19.8) | 1499 (21.7) | 646 (16.3) | 703 (18.0) | 416 (20.8) | 483 (24.0) | 364 (17.9) |
| Anticipated interval for rectal bleeding |  |  |  |  |  |  |  |
| Short | 17634 (84.7) | 5899 (85.5) | 3569 (90.2) | 3200 (81.9) | 1690 (84.5) | 1596 (79.4) | 1680 (82.4) |
| Long (> 1 week) | 2857 (13.7) | 931 (13.5) | 335 (8.5) | 571 (14.6) | 289 (14.5) | 384 (19.1) | 347 (17.0) |
| Recognition of persistent cough or hoarseness as cancer symptom |  |  |  |  |  |  |  |
| Yes | 15155 (72.8) | 4798 (69.5) | 2941 (74.3) | 3002 (76.8) | 1559 (78.0) | 1510 (75.2) | 1345 (66.0) |
| No | 4951 (23.8) | 1783 (25.8) | 867 (21.9) | 831 (21.3) | 402 (20.1) | 436 (21.7) | 632 (31.0) |
| Recognition of unexplained bleeding as cancer symptom |  |  |  |  |  |  |  |
| Yes | 18187 (87.4) | 6192 (89.7) | 3573 (90.3) | 3469 (88.8) | 1580 (79.0) | 1796 (89.4) | 1577 (77.3) |
| No | 2018 (9.7) | 513 (7.4) | 265 (6.7) | 371 (9.5) | 314 (15.7) | 169 (8.4) | 386 (18.9) |
| Number of negative cancer beliefs (coded as) |  |  |  |  |  |  |  |
| 0 (0) | 6427 (30.9) | 2285 (33.1) | 1282 (32.4) | 1152 (29.5) | 483 (24.2) | 628 (31.3) | 597 (29.3) |
| 1 (1) | 8412 (40.4) | 2689 (39.0) | 1566 (39.6) | 1493 (38.2) | 849 (42.5) | 909 (45.3) | 906 (44.4) |
| 2 (2) | 4180 (20.1) | 1346 (19.5) | 767 (19.4) | 807 (20.7) | 470 (23.5) | 376 (18.7) | 414 (20.3) |
| 3 (3) | 1347 (6.5) | 438 (6.4) | 258 (6.5) | 315 (8.1) | 154 (7.7) | 77 (3.8) | 105 (5.2) |
| 4 (> 4) | 354 (1.7) | 107 (1.6) | 65 (1.6) | 112 (2.9) | 36 (1.8) | 18 (0.9) | 16 (0.8) |
| 5 (> 4) | 81 (0.4) | 33 (0.5) | 15 (0.4) | 23 (0.6) | 8 (0.4) | 1 (0.1) | 1 (0.1) |
| 6 (> 4) | 13 (0.1) | 2 (0.0) | 6 (0.2) | 5 (0.1) | 0 (0.0) | 0 (0.0) | 0 (0.0) |
